# Disruption of Placental Homeostasis Leads to Preeclampsia

**DOI:** 10.3390/ijms21093298

**Published:** 2020-05-07

**Authors:** Akitoshi Nakashima, Tomoko Shima, Sayaka Tsuda, Aiko Aoki, Mihoko Kawaguchi, Satoshi Yoneda, Akemi Yamaki-Ushijima, Shi-Bin Cheng, Surendra Sharma, Shigeru Saito

**Affiliations:** 1 Department of Obstetrics and Gynecology, University of Toyama, Toyama 930-8555, Japan; shitoko@med.u-toyama.ac.jp (T.S.); syk3326jp@yahoo.co.jp (S.T.); aikoyuzu8829@yahoo.co.jp (A.A.); mk.sagamore.hill@gmail.com (M.K.); s812yone@med.u-toyama.ac.jp (S.Y.); au@med.u-toyama.ac.jp (A.Y.-U.); s30saito@med.u-toyama.ac.jp (S.S.); 2 Department of Pediatrics, Women and Infants Hospital of Rhode Island, Warren Alpert Medical School of Brown University, Providence, RI 02903, USA; shibin_cheng@brown.edu (S.-B.C.); ssharma@wihri.org (S.S.)

**Keywords:** autophagy, endoplasmic reticulum stress, inflammation, lysosomes, placenta, preeclampsia, protein aggregation, pyroptosis

## Abstract

Placental homeostasis is directly linked to fetal well-being and normal fetal growth. Placentas are sensitive to various environmental stressors, including hypoxia, endoplasmic reticulum stress, and oxidative stress. Once placental homeostasis is disrupted, the placenta may rebel against the mother and fetus. Autophagy is an evolutionally conservative mechanism for the maintenance of cellular and organic homeostasis. Evidence suggests that autophagy plays a crucial role throughout pregnancy, including fertilization, placentation, and delivery in human and mouse models. This study reviews the available literature discussing the role of autophagy in preeclampsia.

## 1. Introduction

Preeclampsia, a major cause of maternal and perinatal morbidity and mortality, is diagnosed in women with hypertension. It occurs after 20 weeks of gestation and is accompanied by the failure of organs such as kidney, liver, or placenta [1]. Failure in the placental functions results in fetal growth restrictions which is included in the definition of preeclampsia. Thus, placenta, one of the major organs that develops after conception, is of equal importance to maternal and fetal health during gestation and is considered one of the maternal organs for this period. Preeclampsia is known as “the disease of theories”, and it can be induced by a number of conditions including hypoxia, systematic inflammation, immunological dysregulation, placental dysfunction, or increasing antiangiogenic factors in the maternal circulation, especially soluble fms-like tyrosine kinase-1 and soluble endoglin, which are mainly produced by the placenta in both rodents and humans [2,3]. Neutralization of these factors could relieve symptoms of preeclampsia in women [4], and aspirin has been shown to reduce the incidence rate of preterm preeclampsia, but not term preeclampsia [5]. The differences in response to aspirin treatment suggest that the etiology of preeclampsia could differ depending on the stage of the pregnancy when it is induced. The most recognized hypothesis regarding the etiology of preeclampsia is the “two stage model” which comprises poor placentation in stage one followed by systemic endothelial dysfunction in stage two. This model explains the differences in pathophysiology for preterm and full-term preeclampsia [6,7]. In addition, growing evidence suggests that it may be possible to predict preeclampsia before 16 weeks of gestation using maternal characteristics including nulliparity, high maternal age, past history of preeclampsia, antiphospholipid antibody syndrome, chronic hypertension, pre-gestational diabetes, the use of assisted reproductive technology, high body mass index or prior placental disruption [8]. There are also various biophysical and biochemical markers including the pulsatility index of the uterine artery, mean arterial pressure, and placental growth factor expression (PlGF) amongst others [9].

We have focused on autophagy as a new cellular mechanism to maintain placental homeostasis [7,10,11]. Cellular homeostasis is maintained by balancing protein synthesis and degradation. Synthesized proteins must be eventually degraded in cells, otherwise excessive aggregated proteins lead to cellular malfunction, a process that can be prevented by autophagy via promoting protein degradation. Protein degradation is facilitated by two pathways: the autophagy-lysosomal system, which mainly targets long-lived proteins, and the ubiquitin-proteasome system, which targets short-lived proteins [12,13,14]. Failures in the autophagy pathways contribute to the development of several human diseases, including neurodegenerative disorders. In this case, misfolded proteins that are not degraded by autophagy accumulate in the central nervous system, inducing neurodegenerative diseases [15]. On the other hand, this process is a vital component in energy production under starvation conditions. Macroautophagy, a non-selective degradation process, is the main physiological process by which autophagosomes, a unique structure of autophagy, deliver their internal contents to the lysosomes to facilitate degradation [16]. By contrast, selective autophagy, including mitophagy (targeting the mitochondria), ER-phagy (endoplasmic reticulum), aggrephagy (protein aggregates), and xenophagy (pathogens), is vital to fundamental cellular regulation. Changes in these pathways may result in cellular stress, immunological response, or tumorigenesis [17]. This review summarizes the role of autophagy in placental homeostasis in the prevention of preeclampsia, a multifactorial disease, from multiple viewpoints. The term “autophagy” refers to “macroautophagy” throughout this manuscript unless otherwise stipulated.

## 2. Autophagy in Preeclampsia

Autophagy is activated by various stimuli, including starvation, hypoxia, infection, and endoplasmic reticulum (ER) or oxidative stress. An isolation membrane generated from the ER-mitochondrial contact site engulfs some organelles, forming a double membrane structure called an autophagosome, and degrades the inner membrane of this construct using autolysosomes, which are a complex of autophagosomes fused with lysosomes [18]. Autophagy is activated during early pregnancy placentation. This was confirmed by studies that demonstrated an increase in microtubule-associated protein 1 light chain 3 beta (MAP1LC3B) dots in the cytoplasm of human extravillous trophoblasts (EVTs), which invade the decidua basalis [19]. Hypoxia, which is a known physiological stress during early pregnancy, induces autophagy activation in primary trophoblast cells in vitro [19,20]. Autophagy was more active in term placentas obtained from cesarean section than in those collected following vaginal delivery [21], irrespective of the mode of labor induction [22]. However, placental autophagy in preeclampsia and gestational diabetes mellitus remains controversial [19,23,24,25,26].

Invasion and vascular remodeling of EVTs is necessary for normal placentation. Impairment of these functions leads to poor placentation during stage one of the “two stage preeclampsia model” [7]. Autophagy is required to reduce galectin-4 expression for normal placental development and the differentiation of invasive trophoblasts in a normal pregnant rat [27,28]. In humans, the activation of autophagy was observed in EVTs, which invaded deeply into decidua [19]. To estimate the correlation between autophagy and EVT functions in humans, autophagy-deficient human EVT cell lines—HTR8-autophagy-related (Atg)4B^C74A^ and HchEpC1b-Atg4B^C74A^ cells, generated by stably expressing Atg4B^C74A^—were constructed. An inactive mutant of Atg4B inhibits autophagic degradation by blocking the lipidation of MAP1LC3B paralogs [29]. Using these cell lines, autophagy deficiency was shown to contribute to the inhibition of invasion and impairment of vascular remodeling under hypoxic conditions in vitro [19], indicating that autophagy sustains EVT functions under hypoxia.

Hypoxia inducible factor1α (HIF1α) is known to be essential for EVT invasion [30]. It is stabilized during hypoxia by inhibiting proteasome-mediated degradation. On the other hand, chaperone-mediated autophagy is also involved in HIF1α degradation in the liver during nutrient deprivation [31,32]. Regarding the role of autophagy in trophoblasts under hypoxia, the invasiveness of autophagy-deficient cells was significantly lower when hyper-expressing HIF1α, as demonstrated by the reduction in adenosine triphosphate (ATP) production in these cells [33]. ATP levels were also significantly decreased in severe preeclamptic placentas when compared to normal placentas [34]. Autophagy suppression was observed in the syncytiotrophoblast layer, which contributes to nutritional and oxygenic exchange between mothers and fetuses in preeclamptic placentas [35]. These results suggested that the impairment of autophagy, including mitophagy, contribute to ATP reduction in placentas under hypoxic stress. As for HIF1α as the treatment target of preeclampsia, hyper-expression of HIF1α by hypoxia/reoxygenation was shown to be reduced by melatonin, which protected the syncytiotrophoblasts by restoring autophagy [36]. Excessive autophagy activation by oxidative stress inhibited EVT invasion in HTR8 cells [37], but apoptosis induced by oxidative stress was rescued following treatment with resveratrol, an activator of autophagy in HTR8 cells [38]. Taken together, the adequate regulation of autophagy via HIF1α modulation might be able to restore trophoblasts’ functions during placentation.

That being said, there is some conflicting data on this subject. Autophagy activation was confirmed by Western blot when researchers observed increases in MAP1LC3-II and decreases in sequestosome 1/p62 (p62) expression in placentas from patients with hypertensive disorder [23], and the presence of autophagic vacuoles in the syncytial layer of preeclamptic placentas [26]. In the case of autophagy activation, ceramide overload, which induces excessive autophagy, might be involved in placental dysfunction in preeclampsia in concert with oxidative stress [39]. In addition, Atg4B overexpression may activate autophagy via the downregulation of microRNA let-7i in preeclamptic placentas [40]. To evaluate the role of autophagy in the pathophysiology of preeclampsia, it would be useful to identify ways to quantify autophagy or to validate the use of specific biological markers that represent specific autophagy responses.

Further analysis was performed using two independent placenta-specific autophagy inhibition mouse models. One was a syncytiotrophoblasts layer-specific Atg7-deficient mouse model, and the other Atg7- was deleted in all the layers of the placenta using a lentivirus system [41,42]. Atg7 mediates autophagy activation via two conjugations of Atg12 to Atg5, and Atg8 to phosphatidylethanolamine like E1-like protein in the ubiquitin-proteasome system [43]. MAP1LC3 proteins, a major marker of autophagy, were repressed in the labyrinth layer when compared to the decidua basalis [22], but no difference was observed in the Atg7 expression levels among the layers in the normal murine placentas [41]. In the Atg7 knockout placenta, the area of the spongiotrophoblast layer, but not the labyrinth layer, was smaller than that of the control placenta, indicating that differentiation and growth of the spongiotrophoblast layer is more sensitive to autophagy deficiency. In the case of the placenta-specific autophagy deficiency models, the syncytiotrophoblasts layer-specific Atg7 deficient mouse showed “fetal” growth restriction. This suggests that autophagy suppression in the syncytiotrophoblasts, which mediate nutritional exchange, affects fetal growth. The mouse with Atg7 deficiency in all layers showed “placental” growth restriction, accompanied with impaired invasion and vascular remodeling of invasive trophoblasts. Autophagy suppression in spongiotrophoblasts or parietal trophoblast giant cells, in which apoptotic cells were increased following Atg7 deletion, might affect placental growth. The depletion of Atg7 elicits p53-dependent apoptosis as a result of the DNA damage caused by nutritional restriction [44]. Thus, autophagy-related cell death may be involved in the fetal and/or placental growth restrictions exhibited by patients with preeclampsia. Kojima et al. estimated that Atg9a, which is involved in autophagosome formation in multiple human organs, in the heterozygous p57^Kip2^ mice (preeclampsia model mice) developed hypertension and proteinuria in dams [45]. The hetero- or homozygous deletion of Atg9a in pups increased the incidence of fetal death, compared to that of the wild type [46]. In addition, the body weights of Atg9a homozygous-deleted pups were significantly lower than those of Atg9a heterozygous-deleted or wild type pups. Taken together, these data suggest that autophagy plays a protective role in preventing the development of preeclampsia. There are many preeclampsia-like mouse models associated with the renin-angiotensin system, ephrin B2, or storkhead box 1 [47,48,49,50,51], but the role of autophagy in these models is entirely unknown. For instance, the overexpression of storkhead box 1, a preeclampsia-related gene, was associated with the hyperactivation of mitochondria, resulting in increased free radicals in the placenta [47]. In response to this change, mitophagy, a selective autophagy of mitochondria to maintain mitochondrial homeostasis, may reduce the production of free radicals in trophoblasts. Using these models, novel functions of autophagy might be discovered in preeclamptic placentas.

Recently, Yin Yang 1, a transcription factor for the cytoskeleton-related proteins whose mRNA level was shown to be lower in recurrent spontaneous abortion patients, has been identified as a regulator of autophagy in trophoblasts [52,53]. In addition, Fetuin-A, an endogenous inhibitor of the insulin receptor, which increases in women with gestational diabetes mellitus, inhibits autophagy via repression of beclin1 (BECN1) [54]. Peptidome analysis revealed that BECN1 is decreased in amniotic fluid from women with preeclampsia [55]. These pregnancy-associated autophagy regulators could explain autophagy inhibition of preeclampsia.

## 3. Lysosomal Dysregulation in Preeclampsia

**Figure 1:**
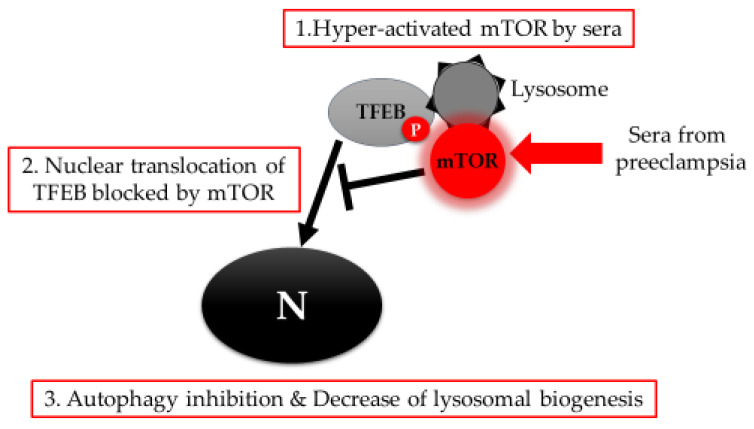
TFEB-mediated autophagy inhibition. Sera from preeclampsia induce hyper-activation of mTOR, which blocks nuclear translocation of TFEB. TFEB inactivation results in the inhibition of autophagy and lysosomal biogenesis in trophoblasts. N: nucleus.

Several neurodegenerative diseases, including Parkinson’s disease, Alzheimer’s disease (AD), and Huntington’s disease, are caused by the deposition of aggregated proteins in the central nervous system. Some of these aggregates are mediated by autophagy inhibition [15,56]. As a result, some work has been conducted to evaluate the possibility that preeclampsia may result from protein aggregation [57,58,59]. There is a report that women with hypertension in pregnancy are at higher risk of mortality as a result of AD [60]. Accumulation of amyloid-β, which causes AD, is prevented by LC3-associated endocytosis mediated by the RUN and cysteine-rich domains of BECN1 interacting protein (Rubicon) and Atg5 in the microglia [61]. In pregnancy, aggregated proteins, including amyloid-β or transthyretin, are detected in the serum and urine samples of preeclampsia patients [57,59], and are deposited with higher frequency in the autophagy-deficient cells [35]. This accumulation also induced preeclampsia-like features in pregnant mice [62]. Thus, the molecular mechanism by which autophagy inhibition leads to the accumulation of aggregated proteins in the placenta must be elucidated so that we can better understand the pathophysiology of preeclampsia. In that regard, we reported that downregulation of transcriptional factor EB (TFEB) with lysosomal dysfunction was a hallmark of preeclamptic placentas [35]. TFEB, a member of the basic Helix-Loop-Helix-Zipper family of transcription factors, is called a master regulator of autophagy and lysosomal biogenesis [63]. During placental development, loss of TFEB leads to severe defects in vascularization due to the loss of vascular endothelial growth factor (VEGF) in the labyrinth layer of these placentas [64]. TFEB is downregulated by hypoxia in primary trophoblasts, and the inhibition of nuclear translocation, a marker of TFEB activation, was mediated by the hyperactivation of the mammalian target of rapamycin (mTOR). Similar associations between hyperactivated mTOR and lysosomal dysfunction disrupted proper function in chondrocytes from the lysosomal storage diseases-mouse model [65]. Interestingly, TFEB downregulation was confirmed in two independent placenta-specific autophagy deficient murine models [42]. This finding suggests that there is an important correlation between TFEB downregulation and autophagy inhibition in preeclamptic placentas. In addition, sera from preeclampsia patients, which inhibited autophagy (unpublished data) via activated mTOR in trophoblast cell lines, partially inhibited the nuclear translocation of TEFB, as shown in Figure 1 [35]. 

The sera from preeclampsia also induce reactive oxygen species production accompanied by mitochondrial swelling [66]. In addition, irrespective of TFEB, rupture of the lysosomes themselves may pose a threat to trophoblast health by releasing hydrolases into the cytoplasm [67], because a lack of lysosomes impairs the step of autophagosome-lysosome fusion. Though autophagy protects cells from the cytotoxicity of microparticles by sequestering these particles in the trophoblasts [68], exposure of mineral crystals, including monosodium urate and silica, or silica nanoparticles, may increase the risk of preeclampsia because they impair lysosomes [69]. The degradation of damaged lysosomes detected by galectin-3 conjugation using autophagy machinery demonstrated that lysophagy retains cellular homeostasis by restoring low pH. Otherwise, continuous lysosomal damage could disrupt lysosomal biogenesis. Placenta produces specific galectins—galectin-13, galectin-14, and galectin-16—encoded in a gene cluster on chromosome 19 that is suspected to evolutionally regulate homeostasis in placentas [70]. The expression of galectin-13 and galectin-14 has been shown to decrease in preterm preeclampsia accompanied by fetal growth restriction [71]. The correlation between placenta-specific galectins and autophagy has not been elucidated yet, which would be worth investigating from the viewpoint of placental homeostasis.

Hyperactivation of AMP-activated protein kinase (AMPK), which induces autophagy, was reported in preeclampsia [72], and AMPK-mediated acetyl-CoA synthetase 2. This is activated by AMPK and binds to TFEB, resulting in autophagy activation and lysosomal biogenesis [73]. Paradoxically, these changes may be derived from TFEB downregulation in preeclamptic placentas. In addition to the dysregulation of TFEB, decreases in p97/Valosin-containing protein, which is a member of the ATPase associated protein family, might mediate autophagy inhibition in preeclamptic placentas [74]. Mutations in the p97/Valosin-containing protein, which is involved in a variety of cellular functions including autophagy, ER stress response, endosomal trafficking, cell cycle, and DNA repair, have also been shown to be related to the development of several degenerative diseases, including frontotemporal dementia and amyotrophic lateral sclerosis [75]. Therefore, the dysfunction of p97/Valosin-containing protein might be worthy of remark on preeclampsia.

## 4. Endoplasmic Reticulum Stress and Autophagy in Preeclampsia

ER stress is believed to contribute to the pathophysiology of preeclampsia [76]. To show the correlation between ER stress and poor placentation in a mouse model, prolonged exposure of tunicamycin, which increases the expression of glucose-regulated protein 78 and C/EBP-homologous protein in the placenta, inhibited the growth of placentas and fetuses [77]. However, moderate ER stress is fundamental to the adaptation of the placenta in early development. This is because inositol requiring enzyme-1 (IRE1), a protein on the ER membrane, is constitutively activated in the placenta, but not in the fetus, and the loss of IRE1α elicited embryonal absorption after 12.5 days of gestation in the IRE1-knockout mice. These placentas also exhibited morphological disruption in the labyrinth layer following a reduction in VEGF-A [78]. Thus, disproportional regulation of ER stress, to a greater or lesser extent, might be related to the development of preeclampsia.

**Figure 2:**
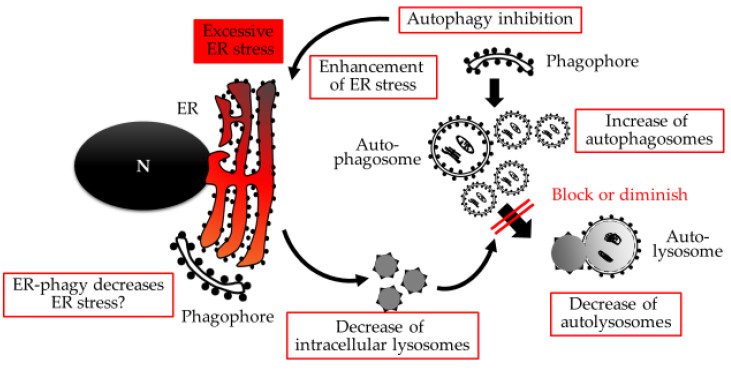
Excessive ER stress-mediated autophagy inhibition. Excessive ER stress, which is enhanced with autophagy inhibition, decreases the number of lysosomes in trophoblasts. The decrease of lysosomes results in the blocking or diminishing of autophagy flux. Meanwhile, ER-phagy might be involved in decreasing ER stress. N: nucleus.

Excessive expression of ER resident proteins binding immunoglobulin protein—PKR-like ER kinase (PERK) and IRE1α, activating transcriptional factor 4 (ATF4) and ATF6—has been described in several reports, and has been linked to early onset preeclampsia [79,80]. ATF4 and ATF6β negatively regulate PlGF secretion in the trophoblasts in response to ER stress via the reduction of PlGF mRNA expression [80]. Decreases in PlGF mRNA were also observed in placenta-specific Atg7 knockout mice [41], suggesting a correlation between autophagy, ER stress, and placental growth via PlGF expression. The morphology of the ER is also affected by preeclampsia with the cisternae of the ER appearing more dilated and the amorphous proteinaceous precipitates fuller in the preeclamptic placenta [76]. Similar dilated ER morphologies were observed in chondrocyte-specific Atg7 deficient mice [81]. Mice without Atg16l1, which is essential for forming autophagosomes via the Atg5-Atg12 complex, exhibited severe colitis like Chron’s disease, accompanied with IRE1α aggregates in the ER, which drive excessive ER stress [82]. In the trophoblast cells, autophagy inhibition by chemical inhibitors increased ER stress, which was confirmed by the increase in immunoglobulin protein expression shown in supplemental Figure 6 of reference 83 [83]. Thus, autophagy could cooperate with ER to maintain cellular homeostasis. In contrast, excessive ER stress decreases lysosomal numbers, which results in the blocking of autophagy flux at the autophagy-lysosomal fusion in trophoblast cells, shown in Figure 2 [83].

The decreased number of lysosomes reflected the decrease in lysosomal-associated membrane protein 1 and beta-galactosidase, a hydrolase in lysosomes, both of which were secreted into the cultured medium. The decreased level of beta-galactosidase was also observed in the sera of women with preeclampsia, suggesting its utility as a marker for detecting ER stress-mediated autophagy inhibition in placentas [83]. IRE1, but not ATF4 or PERK, negatively regulates autophagy by impairing autophagosome assembly, suggesting that IRE1 maintains ER as a scaffold to form the phagophore [84,85]. ER-phagy might be important for dealing with aggregated proteins in the placenta, because ER-phagy prevents the accumulation of aggregation-prone proteins, which link to neurodegenerative diseases using the COPII adaptor complex [86]. Thus, macroautophagy and ER-phagy could be used as measures of protein quality control in human placentas.

## 5. The role of Autophagy for Inflammation in Preeclampsia

**Figure 3:**
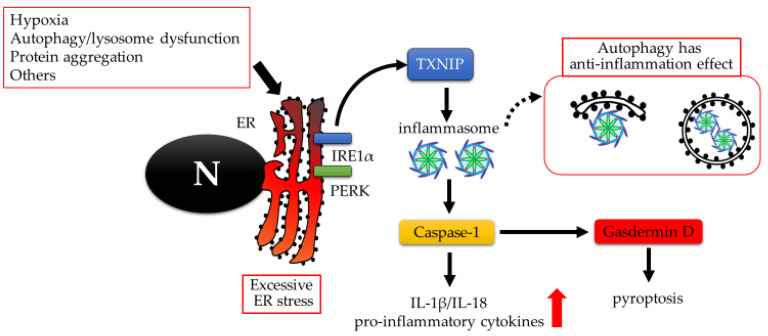
Excessive ER stress-mediated pyroptosis cascade. ER stress activates inflammasomes via Thioredoxin-interacting protein (TXNIP). Activated inflammasome increases the production of active caspase-1, which produces matured form of pro-inflammatory cytokines, IL-18 and IL-1β, and Gasdermin D. Finally, Gasdermin D leads to pyroptosis by forming pores in plasma membranes. Autophagy degrades inflammasome, resulting in preventing inflammation. On the contrary, impaired autophagy may trigger pyroptosis. N: nucleus.

Preeclamptic placentas often involve sterile inflammation. Autophagy has an anti-inflammatory effect, where ubiquitinated inflammasomes marked with p62 are delivered to the autophagosome [87]. Autophagy may also act as an unconventional secretion pathway for the extracellular delivery of inflammasome substrates—interleukin (IL)-1β and IL-18—with lysosomal hydrolase, which is involved in the degradation of autolysosomes [88]. Several types of non-apoptotic cell death, which are related to the inflammation, have recently been proposed, including pyroptosis, ferroptosis, or necroptosis. Though it is not easy to distinguish the non-apoptotic cell death pathways morphologically, the detection of specific molecules in each pathway could allow us to do so. As shown in Figure 3, pyroptosis is confirmed to be associated with elevated levels of active Caspase-1 and its substrate or cleaved products, Gasdermin D, IL-1β, and IL-18. This is not the case for necroptosis, which is characterized by the activation of mixed lineage kinase domain-like proteins with serine358 phosphorylation. Pyroptosis occurs in the placenta during early onset preeclampsia [79]. 

For ferroptosis, preeclamptic placenta tissues showed lower levels of glutathione (an anti-oxidative enzyme which acts to prevent the generation of lipid hydroperoxides caused by reactive oxygen species), glutathione peroxidase activity, and glutathione peroxidase 4 activity than normal placental tissues. This was mediated via miR-30b-5p expression [89]. Interestingly, both pyroptosis, which is accompanied by the induction of NOD-like receptor pyrin-containing receptor 3 (NLRP3), and ferroptosis were induced by hypoxia in trophoblast cells. Especially during pyroptosis, autophagy inhibition of the trophoblast enhanced Gasdermin D expression and the activation of Caspase-1, an inflammasome complex, following their exposure to serum from preeclampsia patients [79]. Similar results were observed in macrophages; the inhibition of autophagy promoted the production and secretion of pro-IL-1β in an NLRP3-dependent manner [90]. Collectively, autophagy activation seems to protect trophoblasts from pyroptosis following exposure to severe hypoxia. In an effort to understand the underlying mechanism of autophagy-mediated protection from pyroptosis, immunity-related GTPase M induces selective degradation of the inflammasome by binding with p62, an autophagy receptor [91]. Treatment with a nonsteroidal hormone, melatonin, may help to protect preeclamptic placentas during hypoxia by activating autophagy and BECN1 protein production.

## 6. Conclusions and Future Directions

Organic homeostasis is maintained by the harmonization of cellular autophagy. Recent studies have shown that autophagy decreases with age, which may explain the increased incidence of neurodegenerative diseases in the elderly, with other studies demonstrating that autophagy dysfunction enhances protein aggregation in the central nervous system. With this in mind, we can assume that aging is also a risk factor in preeclampsia. In addition, evidence suggests that mutations in the Atg genes can result in the development of several human diseases, including Crohn’s disease, Parkinson’s disease, or breast cancer [92]. Though the pathophysiology of preeclampsia is intricately regulated with multiple factors, pathological mutations in Atg genes could be used as a predictive marker for preeclampsia in the near future. The molecular machinery used by the cells to promote autophagy mediates both autophagy-dependent and -independent functions. The non-autophagic functions mediated by the autophagy proteins could be important to maintain proper placental function [93]. Autophagy studies in preeclampsia are underway, and we have a growing understanding of its influence in this pathophysiology. Further studies are needed to clarify the correlation between autophagy and preeclampsia to allow for more robust clinical intervention.

## Abbreviations

AD	Alzheimer’s disease
AMPK	AMP-activated protein kinase
ATF	Activating transcriptional factor
Atg	Autophagy-related
ATP	Adenosine triphosphate
BECN1	Beclin 1
Bip	Binding immunoglobulin protein
ER	Endoplasmic reticulum
EVTs	Extravillous trophoblasts
HIF1α	Hypoxia inducible factor-1α
IL	Interleukin
IRE1	Inositol requiring enzyme-1
MAP1LC3	Microtubule associated protein 1 light chain 3
mTOR	Mammalian target of rapamycin
NLRP3	NOD-like receptor pyrin-containing receptor 3
PERK	PKR-like ER kinase
PlGF	Placental growth factor
Rubicon	RUN and cysteine-rich domain containing beclin1 interacting protein
P62	Sequestosome 1/p62
TFEB	Transcriptional factor EB
VEGF	vascular endothelial growth factor
